# Integrative multi-omics analysis of metabolite–protein interaction networks across different stages of coronary heart disease

**DOI:** 10.1038/s41598-026-66673-0

**Published:** 2026-08-12

**Authors:** Xilun Tan, Yuanxiaoxue Gao, Jia Wang, Xuesen Wang, Yishuang Pan, Mingyu Chen, Meili Gao, Ming Zhang, Chenhao Zhang, Pinsheng Ding

**Affiliations:** Wangjing Hospital of China Academy of Chinese Medical Sciences, Beijing, China

**Keywords:** Biomarkers, Cardiology, Computational biology and bioinformatics, Diseases

## Abstract

To elucidate the molecular characteristics of synergistic interactions across the clinical stages of coronary heart disease (CHD)—specifically stable angina pectoris (SAP), unstable angina pectoris (UAP), and acute myocardial infarction (AMI)—through integrated metabolomic and proteomic analyses. Based on a cohort including SAP, UAP, AMI, and healthy controls, metabolomic and proteomic analyses were performed to identify differentially expressed molecules, followed by KEGG pathway enrichment analysis. Pathways co-enriched across both omics platforms were selected to construct metabolite-protein interaction networks. The number of pathways co-enriched in both metabolomic and proteomic analyses increased markedly with disease stage. Only two pathways (histidine metabolism and arginine and proline metabolism) were identified in the SAP stage; this number increased to five in the UAP stage (including ferroptosis and efferocytosis) and expanded to 25 in the AMI stage, encompassing three major functional modules: immune inflammation, metabolic reprogramming, and cell signaling. The core network exhibited a stepwise increase in connectivity, shifting from a sparse structure in the SAP stage to a highly interconnected architecture in the AMI stage, with L-glutamate and KNG1 identified as the central hubs in this cross-sectional network. In addition, CNDP1 exhibited a stage-dependent functional transition, shifting from downregulation in SAP to upregulation in AMI. In this cross-sectional analysis, metabolic dysregulation and immune activation exhibited stepwise increases in interconnectivity across the SAP, UAP, and AMI groups, with the most extensive crosstalk observed in the AMI stage—a network configuration consistent with a tightly coupled “molecular storm”. These findings provide novel insights into stage-associated molecular signatures of CHD and identify candidate hub molecules for stage-oriented therapeutic investigation.

**Supplementary Information:**

The online version contains supplementary material available at https://doi.org/10.1038/s41598-026-66673-0.

## Introduction

CHD is the leading cardiovascular condition responsible for death and disability worldwide, and its pathological course is characterized by a sequential clinical spectrum from SAP to UAP and ultimately to AMI^1,2^. Although percutaneous coronary intervention and optimized medical therapy have significantly improved patient outcomes, the ability to achieve early identification of and timely intervention in the transition from stable to unstable disease, as well as during acute events, remains limited^3^. This limitation arises because the pathogenesis of CHD is not driven by a linear perturbation of a single molecular pathway but rather by a coordinated cascade involving multiple biological processes, including metabolic reprogramming, activation of immune-inflammatory responses, initiation of cell death programs, and dysregulation of intercellular communication^4^. Traditional single-omics approaches—such as transcriptomics focusing on gene expression, proteomics examining protein abundance, and metabolomics profiling terminal metabolites—can reveal molecular alterations from specific perspectives but are insufficient to capture the systemic patterns of cross-regulation and synergistic dysregulation across multiple molecular layers^5^.

Proteomics and metabolomics occupy the intermediate and downstream levels of biological information flow, respectively. Proteins serve as the primary executors of cellular functions, with their abundance and post-translational modifications governing key processes such as signal transduction, metabolic catalysis, and structural organization. In contrast, metabolites represent the end products of upstream gene-protein regulatory networks, and their levels sensitively reflect the organism’s functional state under physiological and pathological conditions^6^. Integrative analysis of these two omics layers enables the combination of “upstream regulatory” and “downstream functional” information, thereby reconstructing a comprehensive cascade from signal transduction to phenotypic output and offering a unique framework for elucidating the molecular networks underlying complex diseases. In recent years, combined metabolomics-proteomics strategies have advanced significantly in fields such as oncology, neurodegenerative disorders, and metabolic diseases, leading to the identification of key regulatory hubs with both diagnostic and mechanistic relevance^7–9^. However, in CHD research, most existing studies remain limited to cross-sectional analyses at a single omics level or a single disease stage. A comprehensive multi-omics landscape spanning the consecutive clinical stages from SAP to UAP to AMI remains lacking, and the convergence patterns and synergistic interplay between metabolic dysregulation and proteomic regulatory networks across these clinical stages have yet to be fully elucidated^10,11^.

Although our cohort covers the consecutive clinical stages of CHD, the present study employs a cross-sectional design; therefore, our analyses aim to characterize molecular features associated with each clinical stage rather than to trace individual-level disease trajectories. To address this gap, we integrated untargeted metabolomics with tandem mass tag (TMT)-labeled quantitative proteomics data derived from our previously established continuous CHD disease spectrum cohort, including healthy controls, SAP, UAP, and AMI. A dual integration strategy combining pathway enrichment and network construction was employed to systematically characterize the synergistic interactions between the metabolome and proteome across the CHD clinical spectrum. These findings aim to provide new insights into the stage-associated molecular mechanisms underlying CHD from a systems biology perspective and to facilitate the identification of stage-specific biomarkers and therapeutic targets.

## Materials and methods

### Participants

This study was conducted based on a previously established CHD cohort derived from a cross-sectional design. For metabolomic analysis, a total of 250 serum samples were included, comprising 64 healthy controls, 65 patients with SAP, 66 patients with UAP, and 55 patients with AMI. For proteomic analysis, 36 representative serum samples were selected from the same cohort, including 12 healthy controls and 8 patients each with SAP, UAP, and AMI. CHD patients were recruited from the inpatient and outpatient departments of Wangjing Hospital, China Academy of Chinese Medical Sciences, between May 2022 and December 2024, while healthy adult participants were enrolled following confirmation of their health status through physical examination. All CHD patients were classified according to international diagnostic criteria. AMI was diagnosed per the Fourth Universal Definition of Myocardial Infarction^12^, with hs-cTnT > 14 ng/L (99th percentile URL) plus a rise/fall pattern, combined with ECG criteria (ST-segment elevation ≥ 1 mm in limb leads or ≥ 2 mm in precordial leads V2-V3, or non-ST-elevation with dynamic ST-T changes). SAP was diagnosed per the 2024 ESC Chronic Coronary Syndromes Guidelines^13^. UAP was diagnosed per Braunwald classification^14^. All AMI patients were classified as Type 1 MI (spontaneous myocardial infarction due to atherosclerotic plaque rupture) according to the Fourth Universal Definition.

The inclusion criteria were as follows: (1) individuals who met the diagnostic criteria for healthy controls, SAP, UAP, or AMI; and (2) individuals who provided written informed consent and voluntarily participated in the study. The exclusion criteria were: (1) patients with psychiatric disorders; (2) patients with severe hepatic or renal dysfunction; (3) individuals participating in other clinical studies; (4) individuals unable to comply with study procedures; (5) cases with incomplete data that could affect the assessment; and (6) individuals who did not meet the inclusion criteria. This study was conducted in accordance with the latest version of the Declaration of Helsinki and all applicable regulatory requirements. Written informed consent was obtained from all participants prior to enrollment, and the study protocol was approved by an independent ethics committee (No. WJEC-KT-2022-031-P001).

### Serum sample collection and processing

Fasting venous blood was collected on the morning following enrollment. For AMI patients, samples were obtained at a median of 8 h (IQR: 4–12 h) after symptom onset; for UAP patients, 12 h (IQR: 6–24 h) after the most recent episode; for SAP patients, > 48 h after the most recent symptoms. All samples were collected within 30 min of hospital admission and prior to any acute interventions, including antiplatelet loading, anticoagulation, or PCI. SAP samples were collected before morning medications. Chronic medication use was recorded and is presented in Supplementary Table 1.

Fasting venous whole blood (5 mL) was collected from the cubital vein of each participant using serum collection tubes. Blood samples were allowed to stand at 4 °C for 30 min and were subsequently centrifuged at 500 × g for 10 min at room temperature to separate the serum. The supernatant was transferred into 1.5 mL centrifuge tubes and centrifuged again at 13,000 × g for 2 min to remove residual cellular debris. The clarified serum was aliquoted into 1.5 mL screw-cap conical-bottom tubes (200 µL per tube) and stored at -80 °C until batch analysis. Repeated freeze-thaw cycles were strictly avoided throughout collection, processing, and storage.

### Untargeted metabolomics analysis

Serum samples (50 µL) were mixed with 200 µL acetonitrile for protein precipitation and centrifuged at 12,000 rpm for 15 min at 4 °C. The resulting supernatant was collected for analysis. Data acquisition was performed using an Ultimate 3000 ultra-high-performance liquid chromatography (UHPLC) system coupled to an LTQ Orbitrap Velos Pro high-resolution mass spectrometer (Thermo Fisher Scientific, USA). Chromatographic separation was achieved on a Waters UPLC HSS T3 column (1.8 μm, 2.1 mm × 100 mm) with a mobile phase consisting of (A) 0.1% formic acid in water and (B) acetonitrile, delivered at a flow rate of 0.3 mL/min. Mass spectrometry detection was conducted using an electrospray ionization (ESI) source operating in both positive and negative ion modes. The full-scan mass range was m/z 50-2000 with a resolution of 30,000. Quality control (QC) samples were injected every 10 runs to monitor instrument stability.

### Proteomics analysis

Serum samples (100 µL) were subjected to protein extraction using lysis buffer containing 0.1% Triton, and protein concentrations were determined using the BCA assay. Dithiothreitol was added to a final concentration of 10 mM, followed by incubation at 37 °C for 30 min for reduction. Subsequently, iodoacetamide was added to a final concentration of 20 mM, and samples were incubated at room temperature in the dark for 30 min for alkylation. Proteins were purified by methanol-dichloromethane precipitation and digested with trypsin (enzyme-to-protein ratio = 1:50) at 37 °C overnight. The resulting peptides were labeled using a TMT 10-plex kit (Thermo Fisher Scientific). Equal amounts of labeled peptides from each group were pooled and desalted using an Oasis HLB solid-phase extraction cartridge. Peptide separation and detection were performed using an UltiMate 3000 RSLC nanoLC system coupled to an Orbitrap Fusion Lumos Tribrid mass spectrometer. Mobile phase A consisted of 0.1% formic acid in water, and mobile phase B consisted of 0.1% formic acid in 80% acetonitrile, with a 75 min gradient elution. Full MS scans were acquired at a resolution of 60,000 over the m/z range of 350–1200, and MS/MS scans were acquired at a resolution of 50,000 using higher-energy collisional dissociation (HCD) with a normalized collision energy of 32%. Raw data were searched against the UniProt human protein database using Proteome Discoverer 2.4 software, with the false discovery rate (FDR) controlled at ≤ 1%.

From the full cohort of 250 participants, 36 serum samples were selected for proteomic analysis using stratified random sampling by age (≤ 65 vs. >65 years) and sex within each clinical group. Baseline characteristics of the selected subset did not differ from the remaining cohort (all *P* > 0.05). Samples were analyzed using four TMT 10-plex batches (Thermo Fisher Scientific), with channel allocation detailed in Supplementary Table 6. A pooled reference sample, prepared by combining equal aliquots of all 36 individual samples, was included in Channel 131 of every batch. Samples were randomly assigned to channels, with clinical groups evenly distributed across batches. Labeled peptides from each channel were pooled and desalted using an Oasis HLB cartridge. Normalization comprised within-batch total ion intensity normalization, followed by reference channel-based normalization (sample/reference ratio). Between-batch batch effects were corrected using weighted median normalization and the limma “removeBatchEffect” function.

### Differential molecule screening

Principal component analysis (PCA) and orthogonal partial least squares-discriminant analysis (OPLS-DA) were performed using SIMCA 14.1 software. Differentially expressed molecules were screened using thresholds of *P* < 0.05 and either FC > 1.2 (for upregulated molecules) or FC < 1/1.2 (for downregulated molecules). For differential molecule screening, statistical significance was defined as *P* < 0.05 from Student’s t-test or OPLS-DA, without additional multiple-testing correction.

### Multi-omics integrative analysis

Differential metabolites and proteins were mapped to the KEGG pathway database^15^, and pathway enrichment analysis was conducted using MetaboAnalyst 5.0 and the Ouyi Cloud Platform, with *P* < 0.05 considered statistically significant. Shared pathways were defined as KEGG pathways that were significantly enriched (*P* < 0.05) in both the metabolomic dataset and the proteomic dataset for the same pairwise comparison. The intersection of metabolomics-derived pathway lists and proteomics-derived pathway lists was determined using Venn diagrams, and the overlapping pathways were designated as “common interacting pathways”. The number of shared pathways was calculated separately for each of the three comparisons: SAP vs. Health, UAP vs. SAP, and AMI vs. UAP.

Metabolite-protein interaction networks were constructed based on Spearman correlation coefficients calculated between the abundance of differential metabolites and differential proteins within the shared pathways. Only molecule pairs with strong correlations (|r| > 0.6 and *P* < 0.05) were included as edges. Nodes represent metabolites or proteins, and edges represent significant correlations. Networks were visualized using Cytoscape 3.10.4. Hub nodes were identified based on node degree (i.e., the number of edges connected to a node), with the highest-degree nodes designated as central hubs.

### Statistical analysis

Continuous variables are presented as median (first quartile, third quartile), and between-group comparisons were performed using the Kruskal-Wallis test. Categorical variables are presented as number (percentage), and comparisons were performed using the *chi-square* test. Spearman’s rank correlation was used to assess associations between metabolite and protein levels. All statistical tests were two-tailed, with significance set at α = 0.05. Statistical analyses were conducted using SPSS 26.0 and R 4.2.1.

To account for potential confounding by baseline differences among groups, we performed multivariable linear regression analyses for the top 50 differential metabolites and all differentially expressed proteins identified in each comparison. Covariates included age, sex, smoking history, systolic blood pressure, and heart rate—variables that differed significantly among groups at baseline. Stratified sensitivity analyses were performed by sex (male vs. female), age (≤ 65 vs. >65 years), and smoking status (never vs. ever/current) to assess the robustness of the core findings. Interaction terms were tested to evaluate effect modification by each stratification factor.

## Results

### Baseline characteristics of the study population

The baseline demographic and clinical characteristics of the four groups are presented in Supplementary Table 1. Significant differences were observed among the groups in age, sex, heart rate, systolic blood pressure, and smoking history (*P* < 0.05). The median age of each CHD group was higher than that of the healthy control group, and the proportion of males was also greater in the CHD groups. Comparison of baseline characteristics between the 36 proteomic samples and the remaining cohort confirmed no significant differences in age, sex, body mass index, smoking history, or key laboratory parameters (all *P* > 0.05), demonstrating that the proteomic subset was representative of the full study population.

### Differential metabolite screening by metabolomics

**Fig. 1:**
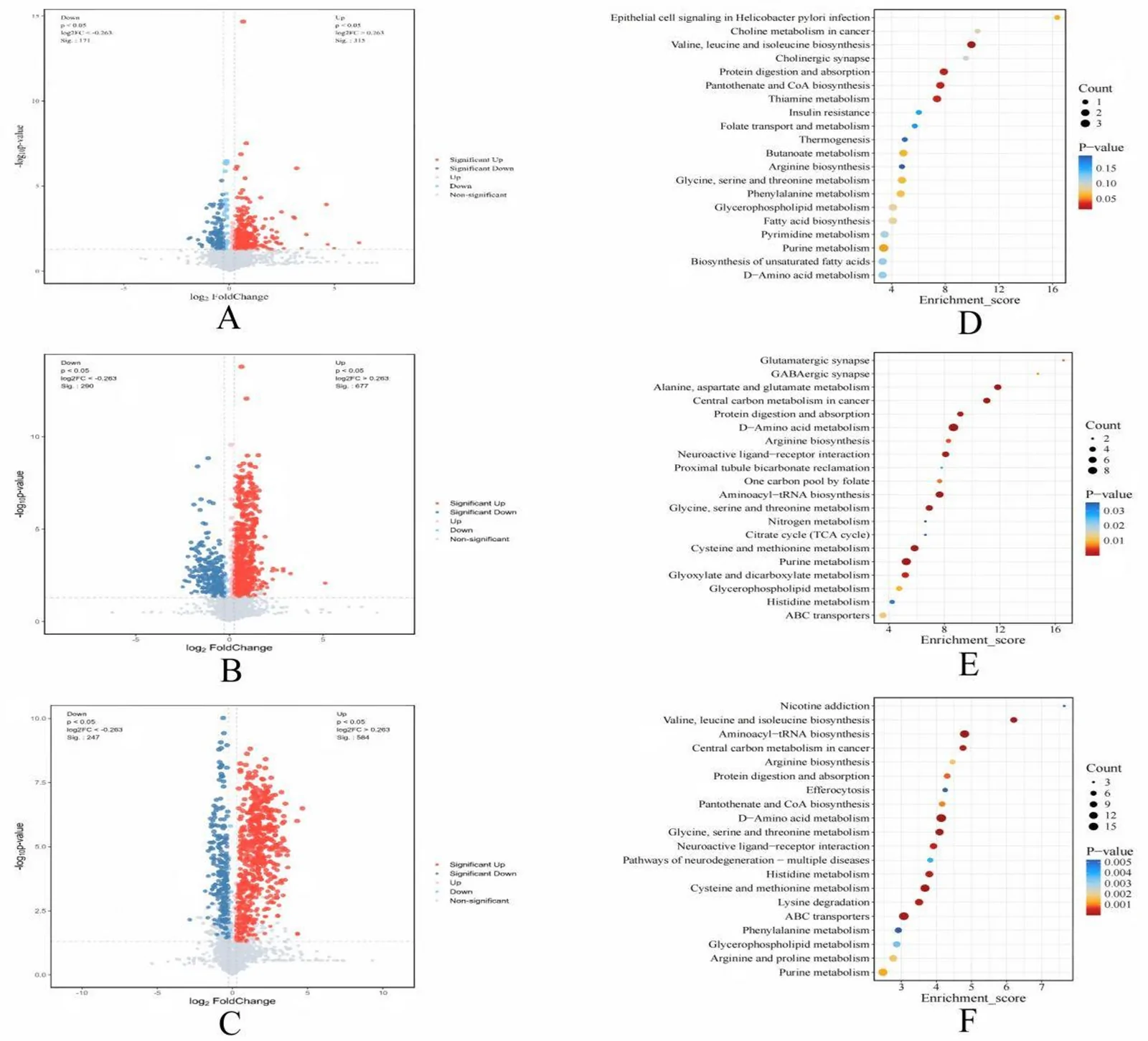
Volcano plots of differential metabolites and KEGG pathway enrichment analysis across different stages of coronary heart disease. (**A**) Volcano plot of differential metabolites for SAP vs. Health. (**B**) Volcano plot of differential metabolites for UAP vs. SAP. (**C**) Volcano plot of differential metabolites for AMI vs. UAP. (**D**) KEGG pathway enrichment analysis of differential metabolites for SAP vs. Health. (**E**) KEGG pathway enrichment analysis of differential metabolites for UAP vs. SAP. (**F**) KEGG pathway enrichment analysis of differential metabolites for AMI vs. UAP. KEGG, reproduced with permission from Kanehisa Laboratories. Source: Kanehisa M, Furumichi M, Tanabe M, Sato Y, Morishima K. KEGG: new perspectives on genomes, pathways, diseases and drugs. Nucleic Acids Res. 2017;45(D1):D353-D361. (https://www.kegg.jp/kegg/kegg1.html).

Following untargeted metabolomic profiling, 486 differential metabolites were identified in the SAP vs. healthy control comparison (Fig. 1A, Supplementary Table 2), 967 in the UAP vs. SAP comparison (Fig. 1B, Supplementary Table 3), and 831 in the AMI vs. UAP comparison (Fig. 1C, Supplementary Table 4). KEGG pathway enrichment analysis of these differential metabolites revealed 46 enriched pathways in the SAP vs. healthy control comparison (Fig. 1D), 79 in the UAP vs. SAP comparison (Fig. 1E), and 149 in the AMI vs. UAP comparison (Fig. 1F), indicating that the extent of metabolic pathway dysregulation showed a stepwise increase across the three clinical groups.

### Differential protein screening by proteomics

**Fig. 2:**
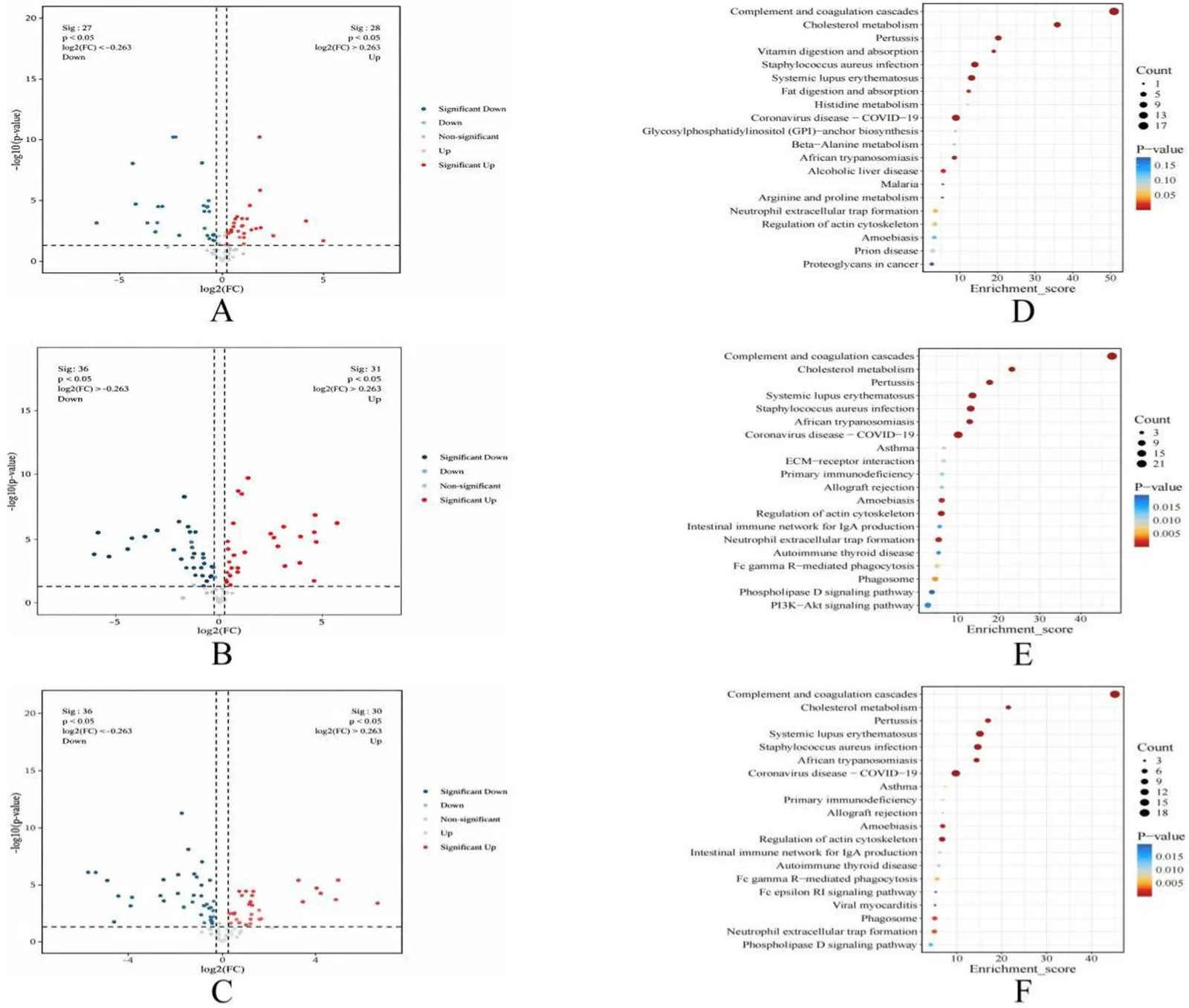
Volcano plots of differential proteins and KEGG pathway enrichment analysis across different stages of coronary heart disease. (**A**) Volcano plot of differential proteins for SAP vs. Health. (**B**) Volcano plot of differential proteins for UAP vs. SAP. (**C**) Volcano plot of differential proteins for AMI vs. UAP. (**D**) KEGG pathway enrichment analysis of differential proteins for SAP vs. Health. (**E**) KEGG pathway enrichment analysis of differential proteins for UAP vs. SAP. (**F**) KEGG pathway enrichment analysis of differential proteins for AMI vs. UAP. KEGG, reproduced with permission from Kanehisa Laboratories. Source: Kanehisa M, Furumichi M, Tanabe M, Sato Y, Morishima K. KEGG: new perspectives on genomes, pathways, diseases and drugs. Nucleic Acids Res. 2017;45(D1):D353-D361. (https://www.kegg.jp/kegg/kegg1.html).

Based on TMT-labeled quantitative proteomics analysis, 55 differentially expressed proteins (DEPs) were identified in the SAP vs. healthy control comparison (Fig. 2A; Table 1), 67 in the UAP vs. SAP comparison (Fig. 2B; Table 2), and 66 in the AMI vs. UAP comparison (Fig. 2C; Table 3). KEGG pathway enrichment analysis of these DEPs identified 56 enriched pathways in the SAP vs. healthy control comparison (Fig. 2D), 70 in the UAP vs. SAP comparison (Fig. 2E), and 72 in the AMI vs. UAP comparison (Fig. 2F). Notably, the complement and coagulation cascades pathway consistently ranked as the most significantly enriched pathway across all three comparisons, representing a core module of protein dysregulation observed across all clinical groups.

**Table 1:** Differentially expressed proteins in the SAP vs. Health comparison.

Accession	Name	Fold Change	log2(FC)	*P*-value
P0DJI8	SAA1	32.5521	5.0247	0.0100
Q06033	ITIH3	17.9646	4.1671	0.0001
P08519	LPA	5.8051	2.5373	0.0034
P02776	PF4	3.8612	1.9491	0.0004
P09871	C1S	3.7835	1.9197	7.16301E-08
P13671	C6	3.6515	1.8685	1.29481E-12
P02671	FGA	3.3483	1.7434	0.0005
P02753	RBP4	2.8125	1.4919	0.0007
P01031	C5	2.6546	1.4085	1.97717E-06
B9A064	IGLL5	2.4146	1.2718	0.0001
P19652	ORM2	2.1476	1.1027	0.0047
Q86YV5	PRAG1	2.1468	1.1022	0.0018
O76096	CST7	2.1348	1.0941	0.0184
P06727	APOA4	2.0660	1.0469	0.0002
P43652	AFM	2.0139	1.0100	4.4374E-05
P80108	GPLD1	1.9643	0.9740	0.0003
P0DOY2	IGLC2	1.8479	0.8859	0.0048
P02748	C9	1.7552	0.8117	0.0010
P05155	SERPING1	1.6953	0.7616	3.11204E-05
P02749	APOH	1.5779	0.6580	0.0001
P02743	APCS	1.5179	0.6021	0.0002
P02649	APOE	1.5146	0.5989	0.0003
P00736	C1R	1.3839	0.4687	0.0007
P04004	VTN	1.3805	0.4652	0.0013
P01008	SERPINC1	1.3030	0.3819	0.0013
P02774	GC	1.2519	0.3241	0.0018
P00751	CFB	1.2217	0.2889	0.0253
P19827	ITIH1	1.2152	0.2812	0.0012
P04114	APOB	0.7801	-0.3583	0.0091
P05546	SERPIND1	0.7704	-0.3764	0.0021
P10909	CLU	0.7659	-0.3847	0.0093
P01011	SERPINA3	0.7624	-0.3914	0.0028
P08697	SERPINF2	0.7449	-0.4249	0.0080
A0A0C4DH25	IGKV3D-20	0.7364	-0.4414	0.0025
P01857	IGHG1	0.6579	-0.6041	1.16076E-05
P27169	PON1	0.6527	-0.6155	6.25956E-07
O75882	ATRN	0.6490	-0.6237	0.0059
P19823	ITIH2	0.6172	-0.6961	0.0034
P0DOX7	/	0.6159	-0.6992	3.57382E-06
P05156	CFI	0.6135	-0.7048	4.36311E-06
P04196	HRG	0.5613	-0.8333	1.03126E-05
P02765	AHSG	0.5592	-0.8386	0.0005
P01834	IGKC	0.5450	-0.8758	2.25012E-06
O14791	APOL1	0.5115	-0.9673	3.041E-10
P55058	PLTP	0.2368	-2.0780	0.0029
Q96PD5	PGLYRP2	0.2086	-2.2610	1.69486E-12
P0C0L4	C4A	0.1909	-2.3889	1.02208E-12
P69905	HBA1	0.1325	-2.9164	3.37508E-06
P29622	SERPINA4	0.1153	-3.1164	3.45419E-06
Q96KN2	CNDP1	0.1114	-3.1668	0.0001
P00748	F12	0.1037	-3.2699	0.0012
P51884	LUM	0.0793	-3.6571	0.0001
P02654	APOC1	0.0539	-4.2139	1.33039E-06
P06681	C2	0.0493	-4.3427	4.1329E-10
A0A0B4J1U7	IGHV6-1	0.0146	-6.0944	0.0001

**Table 2:** Differentially expressed proteins in the UAP vs. SAP comparison.

Accession	Name	Fold Change	log2(FC)	*P*-value
P06681	C2	52.1646	5.7050	3.23892E-08
P51884	LUM	25.9917	4.7000	3.12871E-06
P00748	F12	25.0000	4.6439	5.67071E-09
P29622	SERPINA4	24.3708	4.6071	3.43929E-07
A0A0B4J1U7	IGHV6-1	24.1458	4.5937	0.0099
P55058	PLTP	15.3375	3.9390	8.80277E-07
P02654	APOC1	14.9771	3.9047	0.0003
P04275	VWF	8.9583	3.1632	0.0005
Q96PD5	PGLYRP2	8.5886	3.1024	7.87422E-08
P02750	LRG1	7.2131	2.8506	8.22035E-06
P0C0L4	C4A	6.3665	2.6705	1.39854E-06
P69905	HBA1	5.6386	2.4953	5.34689E-07
P01042	KNG1	2.6635	1.4133	1.56618E-12
P01834	IGKC	2.3464	1.2305	2.8639E-05
P05156	CFI	2.1127	1.0791	8.8311E-11
P19823	ITIH2	1.9047	0.9295	3.54698E-11
Q06033	ITIH3	1.8678	0.9013	0.0008
O75882	ATRN	1.8567	0.8927	0.0017
P04217	A1BG	1.6343	0.7087	0.0001
P19827	ITIH1	1.5911	0.6700	4.03251E-08
P07996	THBS1	1.5147	0.5991	0.0227
P04196	HRG	1.5071	0.5917	0.0009
A0A0B4J1V0	IGHV3-15	1.4611	0.5471	0.0204
P05546	SERPIND1	1.4234	0.5093	0.0037
P02765	AHSG	1.3983	0.4836	0.0002
P00734	F2	1.3328	0.4144	3.12555E-06
P02790	HPX	1.3296	0.4110	1.5537E-05
P10909	CLU	1.2965	0.3746	0.0020
P01011	SERPINA3	1.2960	0.3741	0.0124
P02649	APOE	1.2788	0.3548	0.0145
Q14624	ITIH4	1.2513	0.3234	0.0088
P04004	VTN	0.8077	-0.3081	0.0007
P05155	SERPING1	0.7884	-0.3431	0.0037
P09871	C1S	0.7809	-0.3568	0.0005
P00751	CFB	0.7704	-0.3763	0.0035
P00450	CP	0.7314	-0.4512	0.0047
P01860	IGHG3	0.6497	-0.6222	0.0120
A0A0C4DH25	IGKV3D-20	0.6080	-0.7178	0.0003
P02743	APCS	0.5928	-0.7544	4.22192E-05
P02652	APOA2	0.5915	-0.7576	0.0278
P06396	GSN	0.5866	-0.7696	0.0001
P02775	PPBP	0.5660	-0.8212	0.0038
B9A064	IGLL5	0.5659	-0.8214	0.0008
P02748	C9	0.5403	-0.8881	0.0007
P02774	GC	0.4489	-1.1555	3.67456E-07
P02776	PF4	0.4468	-1.1623	0.0030
P25311	AZGP1	0.4331	-1.2072	3.80292E-05
P08519	LPA	0.4277	-1.2255	0.0239
P02766	TTR	0.4263	-1.2301	0.0007
P01859	IGHG2	0.4098	-1.2872	0.0001
P43652	AFM	0.4090	-1.2899	1.0487E-05
P07357	C8A	0.4014	-1.3169	1.0999E-05
P01031	C5	0.3999	-1.3224	3.4352E-06
P04003	C4BPA	0.3795	-1.3977	3.8049E-07
P06727	APOA4	0.3516	-1.5079	8.95761E-08
P01861	IGHG4	0.3334	-1.5848	0.0007
P13671	C6	0.3207	-1.6406	1.84532E-10
P0DOY2	IGLC2	0.2822	-1.8253	0.0001
P36955	SERPINF1	0.2578	-1.9558	1.95258E-08
A0A0B4J1 × 5	IGHV3-74	0.2155	-2.2141	1.76046E-05
O14791	APOL1	0.1270	-2.9777	1.76914E-07
P80108	GPLD1	0.0846	-3.5630	9.23888E-07
P03952	KLKB1	0.0544	-4.2015	1.34316E-06
P01780	IGHV3-7	0.0481	-4.3781	1.44E-05
P06331	IGHV4-34	0.0258	-5.2770	0.0001
P00736	C1R	0.0178	-5.8125	3.16809E-07
P02671	FGA	0.0158	-5.9846	4.36605E-05

**Table 3:** Differentially expressed proteins in the AMI vs. UAP comparison.

Accession	Name	Fold Change	log2(FC)	*P*-value
P02671	FGA	100.5542	6.6518	0.0001
P00736	C1R	31.5729	4.9806	3.11597E-07
P03952	KLKB1	29.8021	4.8973	4.85462E-05
P80108	GPLD1	19.1313	4.2579	9.55469E-06
Q96KN2	CNDP1	16.7729	4.0681	2.47983E-06
P01780	IGHV3-7	11.1471	3.4786	0.0001
O14791	APOL1	9.7458	3.2848	2.95122E-07
P0DJI8	SAA1	3.2301	1.6916	0.0045
P02652	APOA2	3.0365	1.6024	0.0055
P0DOY2	IGLC2	3.0110	1.5903	0.0006
P36955	SERPINF1	2.5422	1.3461	5.79011E-06
P25311	AZGP1	2.4442	1.2893	1.7169E-05
P01859	IGHG2	2.4357	1.2843	0.0002
P06396	GSN	2.3823	1.2524	0.0018
P06727	APOA4	2.3149	1.2109	0.0051
P01861	IGHG4	2.3134	1.2100	0.0001
A0A0B4J1 × 5	IGHV3-74	2.3055	1.2051	0.0165
A0A0C4DH25	IGKV3D-20	2.2323	1.1585	0.0002
P00739	HPR	2.0312	1.0223	0.0203
P02765	AHSG	2.0115	1.0083	4.87931E-06
P00450	CP	1.7971	0.8457	1.71096E-05
P02775	PPBP	1.6685	0.7386	0.0120
P04003	C4BPA	1.6521	0.7243	5.47696E-06
P02753	RBP4	1.5371	0.6202	0.0060
P02774	GC	1.4280	0.5140	0.0012
P51884	LUM	1.4182	0.5040	0.0016
P27169	PON1	1.2990	0.3774	0.0014
P05546	SERPIND1	1.2673	0.3417	0.0142
Q86YV5	PRAG1	1.2577	0.3308	0.0015
P02790	HPX	1.2033	0.2670	0.0012
P08603	CFH	0.7771	-0.3639	0.0001
P04217	A1BG	0.7679	-0.3811	0.0007
P02749	APOH	0.7676	-0.3815	0.0031
P01834	IGKC	0.7359	-0.4424	0.0149
P43652	AFM	0.7285	-0.4569	0.0093
P02760	AMBP	0.7082	-0.4978	0.0004
P02748	C9	0.7072	-0.4998	0.0034
P07357	C8A	0.7071	-0.5000	0.0063
P19827	ITIH1	0.7012	-0.5121	4.7292E-07
P01031	C5	0.6764	-0.5640	0.0004
P55058	PLTP	0.6269	-0.6738	0.0002
P02763	ORM1	0.5827	-0.7791	0.0125
P0C0L4	C4A	0.5756	-0.7969	0.0002
P00734	F2	0.5514	-0.8587	2.80613E-09
Q96PD5	PGLYRP2	0.5373	-0.8962	1.3936E-06
P10909	CLU	0.5349	-0.9028	2.14774E-05
B9A064	IGLL5	0.5336	-0.9063	0.0019
P09871	C1S	0.4661	-1.1013	1.47523E-07
Q14624	ITIH4	0.4325	-1.2094	5.84566E-08
P04196	HRG	0.4272	-1.2269	1.59858E-05
Q06033	ITIH3	0.4132	-1.2751	0.0001
P05156	CFI	0.3666	-1.4477	1.50137E-10
P07996	THBS1	0.3195	-1.6461	0.0003
P01042	KNG1	0.3018	-1.7285	5.26203E-14
P29622	SERPINA4	0.2683	-1.8983	7.91545E-08
A2NJV5	IGKV2-29	0.2662	-1.9092	9.84273E-06
P05543	SERPINA7	0.1758	-2.5078	0.0001
P13671	C6	0.1749	-2.5157	3.43817E-07
O95445	APOM	0.1606	-2.6384	1.8986E-05
O75882	ATRN	0.0698	-3.8411	2.98357E-05
P02654	APOC1	0.0668	-3.9047	0.0003
P01593	IGKV1D-33	0.0468	-4.4170	2.27262E-05
A0A0B4J1U7	IGHV6-1	0.0414	-4.5937	0.0099
P07225	PROS1	0.0340	-4.8795	4.69454E-07
O76096	CST7	0.0243	-5.3654	3.57026E-08
P06681	C2	0.0192	-5.7050	3.23892E-08

### Stage-associated distribution of common metabolome-proteome interacting pathways across clinical stages

**Fig. 3:**
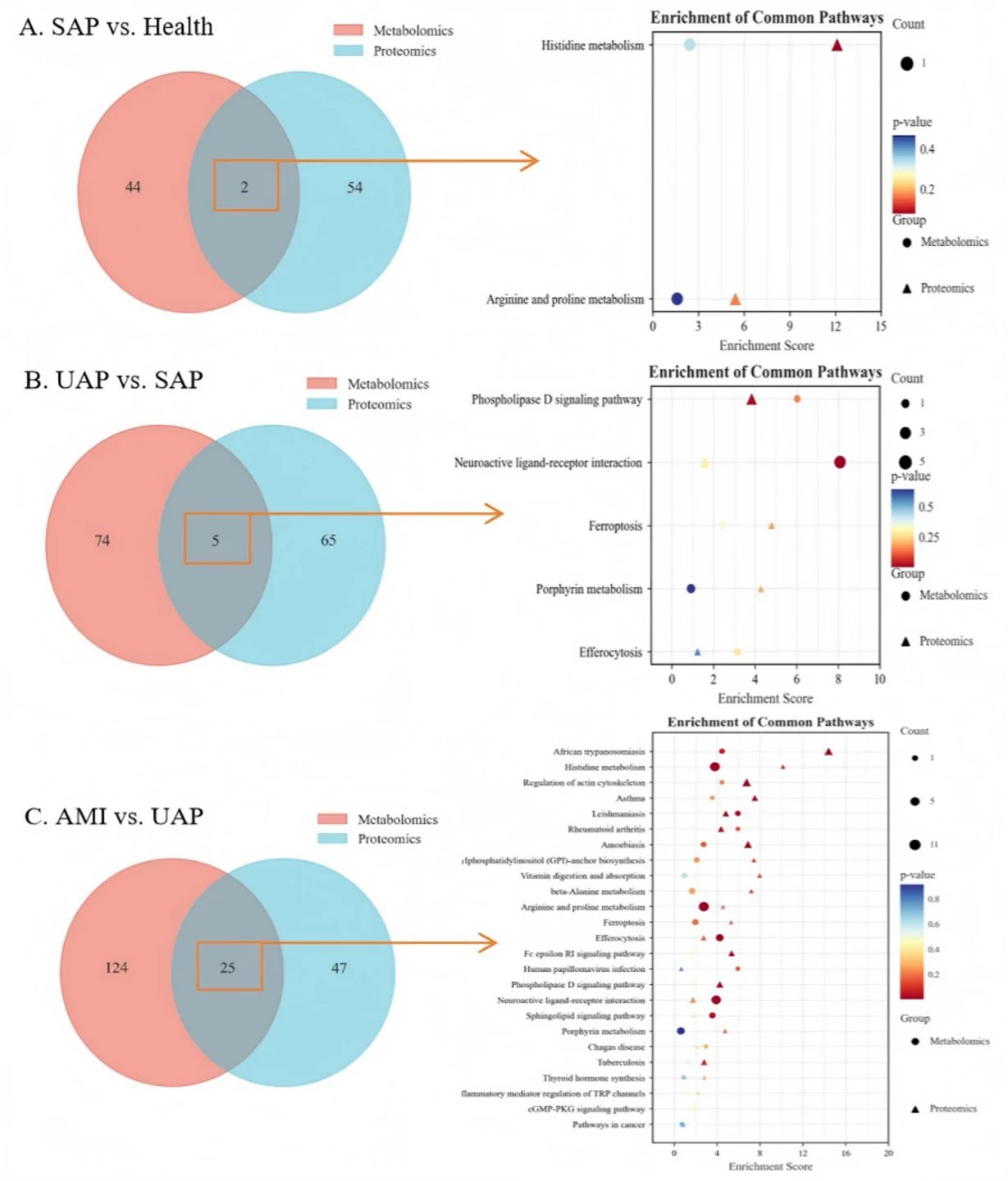
Common KEGG pathway enrichment analysis of metabolomics and proteomics across different stages of coronary heart disease. (**A**) Common pathway enrichment for SAP vs. Health. (**B**) Common pathway enrichment for UAP vs. SAP. (**C**) Common pathway enrichment for AMI vs. UAP. KEGG, reproduced with permission from Kanehisa Laboratories. Source: Kanehisa M, Furumichi M, Tanabe M, Sato Y, Morishima K. KEGG: new perspectives on genomes, pathways, diseases and drugs. Nucleic Acids Res. 2017;45(D1):D353-D361. (https://www.kegg.jp/kegg/kegg1.html).

Venn diagram analysis revealed a marked stage-associated gradient in the number of common interacting pathways shared between the two omics layers: only two common pathways were identified in the SAP vs. healthy control comparison (Fig. 3A), with five identified in the UAP vs. SAP comparison (Fig. 3B), and 25 identified in the AMI vs. UAP comparison (Fig. 3C).

Functionally, the common interactions in the SAP stage were restricted to amino acid metabolism. In the UAP stage, they additionally encompassed immune clearance, programmed cell death, and signal transduction, indicating that synergistic dysregulation between metabolites and proteins was not restricted to basic metabolic processes. In the AMI stage, the 25 shared pathways were classified into three major functional modules—immune inflammation, metabolic reprogramming, and cell signaling and death—reflecting a comprehensive destabilization across multiple biological systems.

### Stage-Specific Topological Features of Core Metabolite-Protein Interaction Networks

Based on correlation analysis between differential metabolites and differentially expressed proteins within the shared pathways, stage-specific interaction networks were constructed. The network structure exhibited a clear pattern of increasing complexity associated with more advanced clinical stages.

**Fig. 4:**
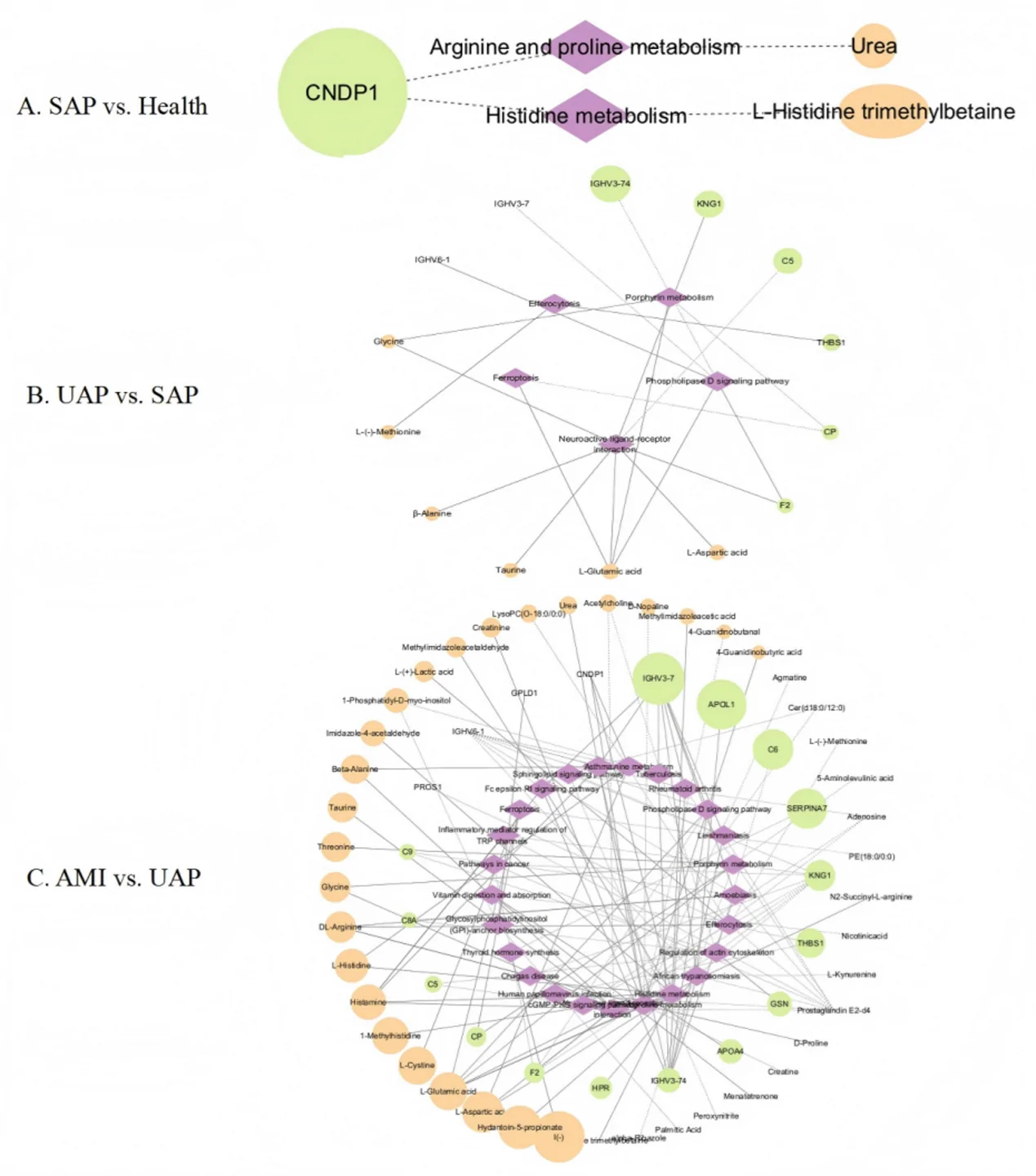
Core regulatory networks of metabolomics and proteomics across different stages of coronary heart disease. (**A**) Core regulatory network for SAP vs. Health. (**B**) Core regulatory network for UAP vs. SAP. (**C**) Core regulatory network for AMI vs. UAP.

In the SAP vs. healthy control comparison, the network was the simplest, involving only 2 pathways, 1 metabolite node, and 1 protein node (Fig. 4A), suggesting limited metabolite-protein interaction at the SAP stage. In the UAP vs. SAP comparison, network complexity increased markedly, encompassing 5 pathways, 6 metabolites, and 8 protein nodes (Fig. 4B), and exhibiting emerging modular features. Amino acids and their derivatives participated in multiple metabolic and signaling pathways, while proteins such as complement components, coagulation factors, and ceruloplasmin were embedded within immune and cell death-related modules; immunoglobulin heavy chain variants also appeared for the first time.In the AMI vs. UAP comparison, network complexity was highest, covering 25 pathways, 45 metabolites, and 19 protein nodes (Fig. 4C), and displayed a highly interconnected architecture. L-glutamate and KNG1 emerged as the hub nodes with the highest degree at the metabolite and protein levels, respectively, each connecting to six pathways. Overall, the network demonstrated a tightly integrated structure in which immune inflammation, metabolic reprogramming, and signal transduction were extensively interwoven.

### Covariate-adjusted analysis of core differential molecules with sex-, age-, and smoking-stratified sensitivity analyses

Covariate-adjusted analyses confirmed that all core hub molecules—including CNDP1, KNG1, L-glutamate, THBS1, and CP—remained significantly associated with CHD stage status after adjustment for age, sex, smoking history, systolic blood pressure, and heart rate (all adjusted *P* < 0.05; Supplementary Table 5). Stratified sensitivity analyses by sex, age, and smoking status revealed no significant effect modification (all *P* for interaction > 0.05; Supplementary Table 5). The key functional pathways also remained significantly enriched after covariate adjustment (all adjusted *P* < 0.05).

## Discussion

This study integrated metabolomic and proteomic data and employed a dual strategy combining pathway enrichment and network construction to systematically characterize the synergistic interaction landscape between the metabolome and proteome across the clinical spectrum of CHD from SAP to UAP to AMI. The main findings can be summarized in three key points. First, the number of common interacting pathways shared between the two omics layers increased markedly from 2 in the SAP stage to 25 in the AMI stage, representing a nonlinear stage-associated gradient. Second, the core interaction network shifted from a simple, sparse structure in the SAP stage to a highly interconnected architecture in the AMI stage, with L-glutamate and KNG1 emerging as central hub nodes. Third, key molecules such as CNDP1 exhibited opposite regulation trends in different comparisons, suggesting stage-associated shifts in their abundance patterns. Collectively, these findings provide new insights into stage-associated molecular signatures of CHD from a systems biology perspective.

The most prominent macroscopic finding of this study is that both the number and functional categories of common interacting pathways shared between the metabolome and proteome differed markedly across the three clinical groups, with a greater number observed in the AMI group compared with the SAP and UAP groups. Only two pathways (histidine metabolism; arginine and proline metabolism) were identified in the SAP stage, with five identified in the UAP stage with the emergence of efferocytosis, ferroptosis, and phospholipase D signaling, and 25 identified in the AMI stage. In the AMI stage, these pathways formed a dense network comprising three major functional modules: immune inflammation, metabolic reprogramming, and cell signaling transduction. This cross-sectional pattern is consistent with a model of systemic destabilization in which metabolic dysregulation and immune activation showed limited interdependence in the SAP group but demonstrated extensive interconnectivity in the AMI group. In the SAP stage, the complement and coagulation cascades were already significantly enriched, indicating a low-grade activation of innate immunity; however, its coupling with metabolic disturbance remained limited, presenting a pattern of “metabolism taking precedence while immune activation remains latent”. This is consistent with the clinically stable presentation and only mildly elevated myocardial injury markers observed at this stage, and aligns with the pathological characteristics of early atherosclerosis, which are dominated by lipid infiltration and endothelial dysfunction^16^. In the UAP group, the involvement of efferocytosis and ferroptosis pathways was observed, suggesting the emergence of immune-metabolic crosstalk at this disease stage. Impaired efferocytosis promotes secondary necrosis of apoptotic cells and the release of damage-associated molecular patterns^17,18^, while ferroptosis contributes directly to plaque necrotic core expansion and instability^19^. The convergence of these processes with metabolic dysregulation provides a systems-level explanation for plaque destabilization. In the AMI group, a highly interconnected network encompassing immune inflammation, metabolic reprogramming, and signal transduction was identified. Compared with previous studies that focused on post-infarction molecular alterations at a single-omics level^20,21^, the integrative perspective of this study reveals a more fundamental phenomenon: In our cross-sectional data, the acute phase of AMI is not merely a global exacerbation of metabolic disorder but rather a “molecular storm” driven by deep coupling between metabolic and immune-protein networks. In this process, immune inflammation drives metabolic reprogramming to meet the energy demands of acute stress, while metabolic dysregulation further amplifies cell death signaling and immune activation by disrupting redox balance and energy supply, a pattern consistent with a self-reinforcing vicious cycle.

CNDP1 was the only protein node in this study that was differentially expressed in both the SAP and AMI stages and showed a reversal in expression direction, being downregulated in the SAP stage and upregulated in the AMI stage. CNDP1 encodes carnosinase 1, an enzyme that specifically hydrolyzes carnosine, a dipeptide with endogenous antioxidant and carbonyl-scavenging properties that may exert cytoprotective effects under chronic ischemic conditions^22^. The downregulation of CNDP1 in the SAP stage may therefore reduce carnosine degradation, thereby helping to preserve endogenous antioxidant capacity, which could be interpreted as a compensatory protective response. In contrast, the upregulation of CNDP1 in the AMI stage may reflect increased amino acid metabolic remodeling under acute stress; however, excessive enzymatic activity could accelerate carnosine depletion and thereby weaken antioxidant buffering capacity^23^. These cross-sectional differences suggest that CNDP1 abundance may differ across pathological stages of CHD, with the direction of change in SAP being opposite to that in AMI. Whether this reflects a stage-associated shift from a potentially protective state to a maladaptive state remains to be tested in longitudinal studies. This stage-associated difference in expression direction provides a conceptual framework for generating hypotheses about stage-dependent molecular functions in chronic adaptation versus acute decompensation and suggests that the therapeutic relevance of CNDP1 may be highly stage-specific and requires further validation. However, it should be emphasized that these interpretations are based on indirect associations between CNDP1 expression changes and carnosine metabolism. Because carnosine and related metabolites were not directly quantified in this study, a causal relationship within the CNDP1-carnosine axis cannot yet be established. Therefore, future studies should simultaneously quantify CNDP1 expression and carnosine levels in independent cohorts and integrate these findings with functional experiments in cellular and animal models to evaluate the robustness and translational potential of this hypothesis.

In the UAP group, the interaction network exhibited clear modular characteristics. In the enrichment-based interaction network, THBS1 was found to be positively associated with the efferocytosis pathway. Based on the existing literature, efferocytosis is known to mediate the clearance of apoptotic cells, and impairment of this process can lead to secondary necrosis and the release of damage-associated molecular patterns, thereby amplifying inflammatory responses^24^. It is therefore plausible to speculate that the observed association of THBS1 with efferocytosis may reflect the presence of apoptotic cell clearance programs in this clinical group; however, whether this association translates into a functional contribution to efferocytosis impairment and subsequent inflammation remains to be experimentally determined. CP (ceruloplasmin) was negatively associated with ferroptosis and porphyrin metabolism, indicating a potential inhibitory role in iron-dependent lipid peroxidation through the maintenance of iron homeostasis^25^. Notably, immunoglobulin heavy chain variants (IGHV3-7, IGHV3-74, and IGHV6-1) were identified within the UAP network. These molecules are closely involved in the phospholipase D signaling pathway, which not only regulates cytoskeletal rearrangement and vesicular trafficking but also directly participates in immune cell activation and migration^26^. The presence of immunoglobulin-related molecules suggests that adaptive immunity, particularly B-cell-mediated immune responses, may be observed in the UAP group.

In the AMI group, KNG1 was identified as one of the most central hub nodes. In the interaction network, KNG1 abundance was negatively associated with six pathways, including neuroactive ligand-receptor interaction, inflammatory mediator regulation of TRP channels, and the cGMP-PKG signaling pathway. Given that changes in protein abundance do not necessarily equate to changes in protein activity, these negative associations may reflect alterations in KNG1 expression that could be consistent with impaired vasodilatory and myocardial protective responses during the acute phase^27^; however, direct functional studies are required to determine whether the observed abundance changes translate into altered KNG1 activity and downstream physiological consequences. As a key metabolite hub, L-glutamate was linked to six pathways, including histidine metabolism, ferroptosis, and the phospholipase D signaling pathway, reflecting its multifaceted roles in the AMI stage, such as providing alternative energy substrates, supplying antioxidant precursors, and regulating ferroptosis^28,29^. The central hub position of L-glutamate within the AMI network suggests that modulation of glutamate metabolism may simultaneously influence energy metabolism, oxidative stress, and cell death processes, highlighting its potential as a multi-target intervention node in AMI.

This study has several limitations. First, the sample size for the proteomics analysis was limited to 36 cases. Although sufficient for preliminary exploration, this may have reduced the statistical power of the integrative network analysis, and some weaker metabolite-protein associations may not have been detected. Second, the study adopted a cross-sectional design. While this approach enabled systematic characterization of multi-omics network features across different disease stages, it precludes the inference of causal temporal relationships among molecular changes. Third, although the screening criteria for differential molecules are widely used thresholds in omics studies, different cut-off settings may have influenced pathway enrichment results and network topology to some extent. Fourth, the core hub molecules identified in this study (e.g., CNDP1, KNG1, and L-glutamate) remain at the level of correlational evidence; their value as biomarkers or therapeutic targets requires further validation in independent clinical cohorts as well as functional verification in cellular and animal models. Fifth, although blood collection was standardized before acute interventions, the inherent variability in symptom-to-sampling times (particularly in AMI patients, IQR: 4–12 h) may have introduced heterogeneity in molecular profiles. Future studies with serial sampling at predefined time points after symptom onset are needed. Sixth, although we performed covariate-adjusted analyses and stratified sensitivity analyses to account for measured confounders—including age, sex, smoking history, systolic blood pressure, and heart rate—we cannot exclude the possibility of residual confounding by unmeasured variables such as diet, physical activity, or genetic background. Furthermore, the modest sample size of the proteomic cohort (*n* = 36) limited our ability to adjust for a larger set of covariates simultaneously without overfitting. Future studies with larger sample sizes and more comprehensive covariate data are needed to confirm the independence of these associations.

## Conclusion

By integrating metabolomics and proteomics, this study has, for the first time, systematically delineated the cross-sectional landscape of synergistic interactions between the metabolome and proteome across the SAP, UAP, and AMI groups. The results demonstrate a stepwise increase in the number of shared interacting pathways, as well as the stage-associated topological features of the core interaction network. In addition, key molecules, including CNDP1, THBS1, CP, KNG1, and L-glutamate, were identified as hub nodes in different clinical groups. Collectively, these findings provide new insights into the systems-level molecular signatures associated with different clinical stages of CHD and lay a theoretical foundation for the development of multi-omics-integrated, stage-associated biomarkers and stage-tailored intervention strategies.

## Supplementary Information

Below is the link to the electronic supplementary material.


Supplementary Material 1


## Data Availability

The data are provided within the manuscript.
