# Supplementary material for: Integrative multi-omics analysis of metabolite–protein interaction networks across different stages of coronary heart disease
Source: Sci Rep. 2026 Aug 12;16:27226. doi: 10.1038/s41598-026-66673-0 (PMC13529740; doi:10.1038/s41598-026-66673-0)
Supplement: Supplementary file 1 [file 41598_2026_66673_MOESM1_ESM.docx]

Supplementary Table 1. Baseline characteristics of the study population.

| **Variable** | **Health(*n*=64)** | **SAP(*n*=65)** | **UAP(*n*=66)** | **AMI(*n* =55)** | ***P*-value** |
| --- | --- | --- | --- | --- | --- |
| Age, years, Median (Q1, Q3) | 49.5(39.3,60.8) | 70.0(56.5,77.0) | 68.0(57.0,73.0) | 64.0(51.0,74.0) | <0.001 |
| Sex |  |  |  |  |  |
| Male, n (%) | 32(50.0%) | 46(70.8%) | 40(60.6%) | 42(76.4%) | 0.013 |
| Female, n (%) | 32(50.0%) | 19(29.2%) | 26(39.4%) | 13(23.6%) | 0.021 |
| Height, cm, Median (Q1, Q3) | 168.0(162.0,172.0) | 170.0(161.0,173.0) | 165.5(157.8,170.0) | 170.0(160.0,174.0) | 0.069 |
| Weight, kg, Median (Q1, Q3) | 67.5(55.5,74.0) | 70.0(60.5,77.5) | 67.0(61.8,76.0) | 70.0(62.0,78.0) | 0.229 |
| Heart rate, bpm, Median (Q1, Q3) | 72.0(68.0,79.5) | 80.0(72.0,86.0) | 76.5(70.8,82.0) | 80.0(70.0,90.0) | 0.002 |
| Systolic blood pressure, mmHg, Median (Q1, Q3) | 126.0(118.0,132.0) | 134.0(124.0,138.5) | 134.0(124.8,151.0) | 130.0(118.0,149.0) | 0.006 |
| Diastolic blood pressure, mmHg, Median (Q1, Q3) | 75.5(68.3,82.0) | 76.0(69.5,84.0) | 77.0(72.0,84.0) | 79.0(72.0,85.0) | 0.186 |
| Pulse, bpm, Median (Q1, Q3) | 72.0(68.0,79.5) | 78.0(69.5,85.0) | 76.0(70.0,82.0) | 80.0(70.0,89.0) | 0.013 |
| Medical history |  |  |  |  |  |
| Previous PCI, n (%) | 0(0.0%) | 35(53.8%) | 13(19.7%) | 9(16.4%) | <0.001 |
| Previous chronic heart failure, n (%) | 0(0.0%) | 2(3.1%) | 20(30.3%) | 3(5.5%) | <0.001 |
| Previous atrial fibrillation, n (%) | 0(0.0%) | 3(4.6%) | 4(6.1%) | 3(5.5%) | 0.220 |
| Previous hypertension, n (%) | 1(1.6%) | 39(60.0%) | 43(65.2%) | 41(74.5%) | <0.001 |
| Previous hyperlipidemia, n (%) | 0(0.0%) | 34(52.3%) | 35(53.0%) | 19(34.5%) | <0.001 |
| Previous diabetes mellitus, n (%) | 4(6.3%) | 21(32.3%) | 28(42.4%) | 27(49.1%) | <0.001 |
| Previous stroke, n (%) | 0(0.0%) | 11(16.9%) | 16(24.2%) | 3(5.5%) | <0.001 |
| Smoking and alcohol history |  |  |  |  |  |
| Smoking history, n (%) | 11(17.2%) | 29(44.6%) | 33(50.0%) | 30(54.5%) | <0.001 |
| Alcohol consumption history, n (%) | 0(0.0%) | 5(7.7%) | 11(16.7%) | 10(18.2%) | 0.003 |
| Laboratory parameters |  |  |  |  |  |
| TC[mmol/L]，M(Q1,Q3) | 4.68(3.90,5.21) | 3.69(3.15,4.18) | 3.68(3.02,4.89) | 4.09(3.37,4.85) | <0.001 |
| TG[mmol/L]，M(Q1,Q3) | 1.45(0.98,1.90) | 1.34(1.10,1.98) | 1.43(1.02,1.69) | 1.36(1.09,1.73) | 0.984 |
| LDL-C[mmol/L]，M(Q1,Q3) | 2.97(2.44,3.65) | 2.29(1.64,2.69) | 2.20(1.71,3.38) | 2.31(1.39,2.91) | <0.001 |
| HDL-C[mmol/L]，M(Q1,Q3) | 1.26(1.07,1.53) | 1.12(1.01,1.28) | 1.12(0.98,1.27) | 0.95(0.86,1.13) | <0.001 |
| HCY[μmol/L]，M(Q1,Q3) | 13.25(11.15,15.90) | 14.36(12.24,16.95) | 14.49(12.79,17.02) | 17.69(13.80,20.18) | 0.001 |
| FGB[mmol/L]，M(Q1,Q3) | 5.34(4.97,5.99) | 5.63(5.03,7.29) | 5.80(5.02,6.90) | 6.40(5.36,8.02) | 0.008 |
| GHB[%]，M(Q1,Q3) | 5.60(5.40,6.00) | 5.90(5.60,6.35) | 5.75(5.18,6.60) | 5.90(5.50,7.10) | 0.005 |
| ALT[U/L]，M(Q1,Q3) | 19.20(13.63,26.55) | 21.20(13.80,32.75) | 20.05(12.75,28.15) | 25.10(17.40,45.50) | 0.035 |
| AST[U/L]，M(Q1,Q3) | 18.70(15.60,25.48) | 20.10(15.85,27.95) | 21.50(16.00,32.95) | 32.30(21.30,79.20) | <0.001 |
| UA[μmol/L]，M(Q1,Q3) | 304.5(258.8,369.5) | 321.0(270.0,380.0) | 346.0(287.0,402.3) | 342.0(278.0,416.0) | 0.232 |
| Cr[μmol/L]，M(Q1,Q3) | 69.5(60.3,83.0) | 79.0(64.0,90.5) | 75.5(63.0,83.8) | 76.0(64.0,89.0) | 0.077 |
| BUN[mmol/L]，M(Q1,Q3) | 5.00(4.20,5.98) | 5.70(4.55,7.05) | 5.20(4.58,6.43) | 5.00(4.20,6.30) | 0.170 |
| HscTnT[ng/mL]，M(Q1,Q3) | 0.005(0.003,0.008) | 0.012(0.006,0.026) | 0.026(0.014,0.040) | 0.755(0.142,1.700) | <0.001 |
| CK-MB[ng/mL]，M(Q1,Q3) | 1.39(1.03,1.71) | 1.51(1.20,1.83) | 1.74(1.59,2.18) | 68.71(30.26,102.60) | <0.001 |
| Medication use |  |  |  |  |  |
| Antiplatelet agents, n (%) | 0 (0.0%) | 52 (80.0%) | 55 (83.3%) | 27 (49.1%) | <0.001 |
| Statins, n (%) | 0 (0.0%) | 49 (75.4%) | 52 (78.8%) | 24 (43.6%) | <0.001 |
| β-blockers, n (%) | 0 (0.0%) | 38 (58.5%) | 42 (63.6%) | 20 (36.4%) | <0.001 |
| ACE inhibitors/ARBs, n (%) | 1(1.6%) | 35 (53.8%) | 38 (57.6%) | 22 (40.0%) | <0.001 |
| Oral hypoglycemic agents, n (%) | 4(6.3%) | 18 (27.7%) | 27 (40.9%) | 24 (43.6%) | <0.001 |

Continuous variables are presented as median (first quartile, third quartile) [M (Q1, Q3)]. Categorical variables are presented as number (percentage) [n (%)].

Supplementary Table 2. Differential metabolites for SAP vs. Health (top 25).

| **Name** | **Formula** | **m/z** | **RT [min]** | ***P*-value** | **Fold change** | **log2(FC)** | **VIP pred** | **∣log2FC∣** |
| --- | --- | --- | --- | --- | --- | --- | --- | --- |
| Dehydro-4-methoxycyclobrassinin | C_12_H_10_N_2_OS_2_ | 261.01409 | 1.813 | 0.0211 | 71.8290 | 6.1665 | 1.5291 | 6.1665 |
| Moxifloxacin | C_21_H_24_FN_3_O_4_ | 402.18202 | 3.181 | 0.0450 | 31.6050 | 4.9821 | 1.2707 | 4.9821 |
| Elymoclavine | C_16_H_18_N_2_O | 255.1489 | 2.926 | 0.0267 | 25.6304 | 4.6798 | 1.4490 | 4.6798 |
| Clopidogrel carboxylic acid | C_15_H_14_ClNO_2_S | 308.05026 | 3.326 | 0.0001 | 24.4986 | 4.6146 | 2.5926 | 4.6146 |
| 3,5-Dichloro-2-hydroxybenzenesulfonic acid | C_6_H_4_Cl_2_O_4_S | 240.9123 | 2.04 | 0.0070 | 12.6832 | 3.6648 | 2.1244 | 3.6648 |
| Pantoprazole Sulfone | C_16_H_15_F_2_N_3_O_5_S | 398.06058 | 1.876 | 0.0450 | 10.8093 | 3.4342 | 1.4277 | 3.4342 |
| Chloro-methyl-[1-(2-methyl-5,6-dihydro-[1,4]oxathiin-3-yl)-methanoyl]-amino-benzoic acid, isopropyl ester | C_17_H_20_ClNO_4_S | 370.08545 | 1.922 | 8.8225e-007 | 9.1948 | 3.2008 | 3.2278 | 3.2008 |
| 5-Deoxy-5-(dimethylarsoryl)pentonic acid | C_7_H_15_AsO_6_ | 271.01475 | 2.107 | 0.0008 | 8.6390 | 3.1109 | 2.2426 | 3.1109 |
| 3,5-Pyridinedicarboxylic acid, 1,4-dihydro-2,4,6-trimethyl-, diethyl ester | C_14_H_21_NO_4_ | 268.15314 | 8.353 | 0.0007 | 8.3368 | 3.0595 | 2.4123 | 3.0595 |
| Sodium Tetradecyl Sulfate | C_14_H_30_O_4_S | 293.17823 | 10.011 | 0.0467 | 6.0472 | 2.5963 | 1.4504 | 2.5963 |
| 2-Chloro-6-fluorophenol | C_6_H_4_ClFO | 147.00194 | 2.111 | 0.0003 | 5.5825 | 2.4809 | 2.4153 | 2.4809 |
| 2-Carboxy-2,3-dihydro-5,6-dihydroxyindole | C_9_H_9_NO_4_ | 194.04526 | 2.698 | 0.0238 | 5.5171 | 2.4639 | 1.5743 | 2.4639 |
| (E)-4-hydroxyphenylacetaldehydeoxime | C_8_H_9_NO_2_ | 150.05574 | 2.702 | 0.0237 | 5.5042 | 2.4605 | 1.5706 | 2.4605 |
| 2-Hydroxyhippuric acid | C_9_H_9_NO_4_ | 194.04541 | 2.261 | 0.0244 | 5.0413 | 2.3338 | 1.5072 | 2.3338 |
| Pantoprazole sulfide | C_16_H_15_F_2_N_3_O_3_S | 368.08565 | 3.549 | 0.0200 | 4.8042 | 2.2643 | 1.4543 | 2.2643 |
| 2,4-Dichlorophenol | C_6_H_4_Cl_2_O | 160.95604 | 2.041 | 0.0108 | 4.7973 | 2.2622 | 2.0336 | 2.2622 |
| 4-Methyl-5-(2-phosphoethyl)-thiazole | C_6_H_10_NO_4_PS | 222.00087 | 2.09 | 0.0049 | 4.7810 | 2.2573 | 1.8972 | 2.2573 |
| O-Methyl hydrogen phenylphosphonothioate | C_7_H_9_O_2_PS | 189.01221 | 1.976 | 0.0196 | 4.6511 | 2.2176 | 1.4754 | 2.2176 |
| Malyngic acid | C_18_H_32_O_5_ | 329.22904 | 5.609 | 0.0129 | 4.3381 | 2.1171 | 1.5524 | 2.1171 |
| Salicylic acid | C_7_H_6_O_3_ | 137.02408 | 2.098 | 0.0084 | 4.1266 | 2.0450 | 1.7518 | 2.0450 |
| 2,5-Dihydroxybenzaldehyde | C_7_H_6_O_3_ | 137.02411 | 2.771 | 0.0105 | 4.1213 | 2.0431 | 1.7790 | 2.0431 |
| TRIFLUOROALANINE | C_3_H_4_F_3_NO_2_ | 142.01176 | 2.13 | 0.0008 | 4.1004 | 2.0358 | 2.2588 | 2.0358 |
| Fluroxene | C_4_H_5_F_3_O | 125.02172 | 2.128 | 0.0037 | 4.0502 | 2.0180 | 2.0693 | 2.0180 |
| Stearic Acid | C_18_H_36_O_2_ | 283.2633 | 9.436 | 0.0497 | 4.0464 | 2.0166 | 1.4346 | 2.0166 |
| Methyl bis(4-nitrophenyl) phosphate | C_13_H_11_N_2_O_8_P | 355.03516 | 1.973 | 0.0498 | 3.8607 | 1.9489 | 1.2850 | 1.9489 |

Supplementary Table 3. Differential metabolites for UAP vs. SAP (top 25).

| **Name** | **Formula** | **m/z** | **RT [min]** | ***P*-value** | **Fold change** | **log2(FC)** | **VIP pred** | **∣log2FC∣** |
| --- | --- | --- | --- | --- | --- | --- | --- | --- |
| 5-(3-(4-(2-(4-Fluorophenyl)ethoxy)phenyl)propyl)furan-2-carboxylic acid | C_22_ H_21_ F O_4_ | 369.14986 | 2.221 | 0.0083 | 34.6913 | 5.1165 | 1.3223 | 5.1165 |
| Stearamide | C_18_ H_37_ N O | 284.2934 | 14.417 | 0.0025 | 9.6458 | 3.2699 | 1.4734 | 3.2699 |
| Tridemorph | C_19_ H_39_ N O | 298.30884 | 11.898 | 0.0018 | 7.3225 | 2.8723 | 1.4683 | 2.8723 |
| Hexadecanamide;Palmiticamide | C_16_ H_33_ N O | 256.26219 | 12.436 | 0.0014 | 7.3050 | 2.8689 | 1.5469 | 2.8689 |
| N(6)-(Octanoyl)lysine | C_14_ H_28_ N_2_ O_3_ | 273.21614 | 2.928 | 0.0016 | 5.7569 | 2.5253 | 1.4337 | 2.5253 |
| (S)-3-[(Cyanophenylmethyl)amino]-3-oxopropanoic acid | C_11_ H_10_ N_2_ O_3_ | 217.06262 | 15.772 | 0.0132 | 0.1773 | -2.4959 | 1.1284 | 2.4959 |
| diazaphospholane | C_2_ H_7_ N_2_ P | 89.0281 | 2.952 | 0.0059 | 0.1910 | -2.3882 | 1.3132 | 2.3882 |
| Icosanamide | C_20_ H_41_ N O | 312.32448 | 12.821 | 0.0013 | 5.0127 | 2.3256 | 1.5392 | 2.3256 |
| 7-(3-Isothiocyanatopyrrolidin-1-yl)-N,N-dimethyl-2,1,3-benzoxadiazole-4-sulfonamide | C_13_ H_15_ N_5_ O_3_ S_2_ | 352.0555 | 2.236 | 0.0052 | 4.9579 | 2.3097 | 1.3750 | 2.3097 |
| Urolithin a 3-sulfate | C_13_ H_8_ O_7_ S | 306.98931 | 18.053 | 0.0066 | 0.2033 | -2.2983 | 1.2328 | 2.2983 |
| (1Z)-N-[(3Z)-3-(Heptylimino)-3-hydroxypropyl]-2,4-dihydroxy-3,3-dimethylbutanimidic acid | C_16_ H_32_ N_2_ O_4_ | 317.24183 | 2.972 | 0.0018 | 4.8699 | 2.2839 | 1.4108 | 2.2839 |
| (R)-Malate | C_4_ H_6_ O_5_ | 133.01364 | 10.443 | 0.0012 | 0.2085 | -2.2619 | 1.5380 | 2.2619 |
| cis-Aconitic acid | C_6_ H_6_ O_6_ | 173.00848 | 8.937 | 0.0003 | 0.2153 | -2.2153 | 1.6800 | 2.2153 |
| 1H,3H-Thiazolo(3,4-a)benzimidazole, 1-(2,6-difluorophenyl)- | C_15_ H_10_ F_2_ N_2_ S | 287.04769 | 15.518 | 0.0070 | 0.2263 | -2.1439 | 1.2494 | 2.1439 |
| 5-Methylcytidine | C_10_ H_15_ N_3_ O_5_ | 256.09216 | 16.461 | 0.0016 | 0.2294 | -2.1242 | 1.4524 | 2.1242 |
| Febuxostat | C_16_ H_16_ N_2_ O_3_ S | 315.0789 | 15.103 | 0.0031 | 0.2315 | -2.1109 | 1.3822 | 2.1109 |
| Chlorphoxim | C_12_ H_14_ Cl N_2_ O_3_ P S | 331.01112 | 15.518 | 0.0036 | 0.2414 | -2.0507 | 1.3533 | 2.0507 |
| DEOXY-DIFLUOROMETHELENE-PHOSPHOTYROSINE | C_10_ H_12_ F_2_ N O_5_ P | 294.03595 | 15.982 | 5.14883e-005 | 0.2417 | -2.0487 | 1.8918 | 2.0487 |
| Erucin | C_6_ H_11_ N S_2_ | 160.02597 | 4.099 | 0.0282 | 0.2454 | -2.0268 | 1.0754 | 2.0268 |
| Clopidogrel carboxylic acid | C_15_ H_14_ Cl N O_2_ S | 308.04912 | 8.14 | 0.0142 | 4.0452 | 2.0162 | 1.2587 | 2.0162 |
| Armstrong’s acid | C_10_ H_8_ O_6_ S_2_ | 286.96856 | 13.594 | 0.0001 | 0.2474 | -2.0153 | 1.8400 | 2.0153 |
| Ethofumesate | C_13_ H_18_ O_5_ S | 285.08001 | 15.916 | 0.0022 | 0.2532 | -1.9819 | 1.4199 | 1.9819 |
| 2-Amino-6-(bromomethyl)-4(1H)-quinazolinone | C_9_ H_8_ Br N_3_ O | 251.97777 | 15.531 | 0.0001 | 0.2540 | -1.9772 | 1.8255 | 1.9772 |
| (2-Methylphenyl) Dihydrogen Phosphate | C_7_ H_9_ O_4_ P | 187.01748 | 4.012 | 0.0090 | 3.8972 | 1.9624 | 1.1956 | 1.9624 |
| Bctc | C_20_ H_25_ Cl N_4_ O | 371.16385 | 15.976 | 0.0039 | 0.2607 | -1.9396 | 1.3319 | 1.9396 |

Supplementary Table 4. Differential metabolites for AMI vs. UAP (top 25).

| **Name** | **Formula** | **m/z** | **RT [min]** | ***P*-value** | **Fold change** | **log2(FC)** | **VIP pred** | **∣log2FC∣** |
| --- | --- | --- | --- | --- | --- | --- | --- | --- |
| (2-Naphthalenyloxy)acetic acid | C_12_ H_10_ O_3_ | 201.057 | 2.339 | 3.16348e-007 | 25.01582251 | 4.644768983 | 2.43603 | 4.6448 |
| sn-glycero-3-Phosphoethanolamine | C_5_ H_14_ N O_6_ P | 214.04749 | 16.379 | 0.0258999 | 20.00320051 | 4.322158945 | 1.32501 | 4.3222 |
| Amifostine | C_5_ H_15_ N_2_ O_3_ P S | 213.04794 | 16.406 | 0.0244439 | 19.96346686 | 4.319290376 | 1.32775 | 4.3193 |
| DL-Erythrono-1,4-lactone;Erythrono-1,4-lactone | C_4_ H_6_ O_4_ | 117.01934 | 2.613 | 9.9203e-007 | 19.64733042 | 4.296261394 | 2.27661 | 4.2963 |
| (E)-4-hydroxyphenylacetaldehydeoxime | C_8_ H_9_ N O_2_ | 150.05584 | 2.261 | 6.48261e-005 | 12.7721098 | 3.674924956 | 1.67724 | 3.6749 |
| (S)-2-Acetolactate | C_5_ H_8_ O_4_ | 131.03475 | 2.405 | 1.46111e-005 | 12.60635032 | 3.656078755 | 1.8823 | 3.6561 |
| O-Desmethylnaproxen | C_13_ H_12_ O_3_ | 215.0723 | 2.343 | 2.14354e-005 | 12.41172163 | 3.63363134 | 1.97167 | 3.6336 |
| Febuxostat | C_16_ H_16_ N_2_ O_3_ S | 315.0789 | 15.103 | 2.10369e-007 | 11.43731607 | 3.515676638 | 2.24847 | 3.5157 |
| Bctc | C_20_ H_25_ Cl N_4_ O | 371.16385 | 15.976 | 0.000197791 | 11.34903737 | 3.504498028 | 1.62446 | 3.5045 |
| (S)-3-[(Cyanophenylmethyl)amino]-3-oxopropanoic acid | C_11_ H_10_ N_2_ O_3_ | 217.06262 | 15.772 | 1.2189e-005 | 11.18109233 | 3.482989233 | 2.0765 | 3.4830 |
| Iodide Ion | I(-) | 126.90465 | 11.639 | 1.05e-006 | 10.70013097 | 3.41955655 | 2.18934 | 3.4196 |
| Plumbagin | C_11_ H_8_ O_3_ | 187.04162 | 2.393 | 6.37644e-007 | 10.59680125 | 3.405556933 | 2.24582 | 3.4056 |
| 4,7-dioxo-octanoic acid | C_8_ H_12_ O_4_ | 171.06603 | 2.339 | 5.75031e-006 | 10.57651527 | 3.402792464 | 2.2279 | 3.4028 |
| 1H,3H-Thiazolo(3,4-a)benzimidazole, 1-(2,6-difluorophenyl)- | C_15_ H_10_ F_2_ N_2_ S | 287.04769 | 15.518 | 0.000794232 | 10.29277807 | 3.36356052 | 1.50475 | 3.3636 |
| 1-(4-Nitrobenzyl)pyridinium | C_12_ H_11_ N_2_ O_2_ | 214.07594 | 2.341 | 1.10105e-005 | 10.18411868 | 3.348249232 | 2.02934 | 3.3482 |
| 2-methyl-2Z-hexenoic acid | C_7_ H_12_ O_2_ | 127.0764 | 2.326 | 4.65813e-006 | 10.14904893 | 3.343272634 | 2.22461 | 3.3433 |
| Bis(2-cyanoethyl) phosphoramidoite | C_6_ H_10_ N_3_ O_2_ P | 186.04525 | 2.386 | 1.20988e-006 | 10.0617692 | 3.330812097 | 2.19385 | 3.3308 |
| 2-Hydroxyhippuric acid | C_9_ H_9_ N O_4_ | 194.04541 | 2.261 | 1.42518e-005 | 9.908151436 | 3.308615919 | 1.84163 | 3.3086 |
| 6-Carboxy-5,6,7,8-tetrahydropterin | C_7_ H_9_ N_5_ O_3_ | 210.06397 | 15.97 | 0.000203379 | 9.619361852 | 3.265941189 | 1.73502 | 3.2659 |
| 1-Hydroxy-2-phenyl-1,4-dihydro-2,4,1-benzodiazaborinin-3(2H)-one | C_13_ H_11_ B N_2_ O_2_ | 237.08268 | 15.971 | 0.000266139 | 9.566814633 | 3.258038645 | 1.63945 | 3.2580 |
| (R)-Lactate | C_3_ H_6_ O_3_ | 89.02436 | 2.367 | 7.4539e-008 | 9.544990312 | 3.254743733 | 2.34875 | 3.2547 |
| cis-Aconitic acid | C_6_ H_6_ O_6_ | 173.00848 | 8.937 | 0.000223239 | 9.153318078 | 3.194294815 | 1.42896 | 3.1943 |
| 3,4-Dihydro-2H-pyrimido[1,2-c][1,3]benzothiazin-6-imine | C_11_ H_11_ N_3_ S | 216.05891 | 17.146 | 7.29394e-006 | 9.059037749 | 3.179357815 | 1.99502 | 3.1794 |
| Ocfentanil | C_22_ H_27_ F N_2_ O_2_ | 369.1957 | 16.731 | 2.07633e-005 | 8.917981326 | 3.156717179 | 1.98572 | 3.1567 |
| (E)-Glutaconate | C_5_ H_6_ O_4_ | 129.01892 | 8.94 | 0.00194337 | 8.499354049 | 3.087353201 | 1.17071 | 3.0874 |

Supplementary Table 5. Covariate-Adjusted Analysis of Core Differential Molecules with Sex-, Age-, and Smoking-Stratified Sensitivity Analyses

| Comparison | Molecule | Adjusted β (95% CI) | Adjusted *P* | Sex-stratified β (95% CI) | | *P* for interaction (sex) | Age-stratified β (95% CI) | | *P* for interaction (age) | Smoke-stratified β (95% CI) | | *P* for interaction (Smoke) |
| --- | --- | --- | --- | --- | --- | --- | --- | --- | --- | --- | --- | --- |
|  |  |  |  | Male | Female |  | ≤65 years | >65 years |  | Never Smokers | Ever/Current Smokers |  |
| SAP vs. Health | CNDP1 | -2.89 (-4.12, -1.66) | 0.003 | -2.91 (-4.18, -1.64) | -2.84 (-4.21, -1.47) | 0.891 | -2.92 (-4.20, -1.64) | -2.85 (-4.16, -1.54) | 0.872 | -2.87 (-4.15, -1.59) | -2.91 (-4.22, -1.60) | 0.912 |
|  | L-Glutamate | 1.52 (0.89, 2.15) | <0.001 | 1.55 (0.90, 2.20) | 1.48 (0.82, 2.14) | 0.823 | 1.50 (0.85, 2.15) | 1.54 (0.88, 2.20) | 0.845 | 1.49 (0.83, 2.15) | 1.55 (0.89, 2.21) | 0.801 |
|  | SAA1 | 3.12 (1.45, 4.79) | 0.002 | 3.20 (1.48, 4.92) | 2.98 (1.20, 4.76) | 0.742 | 3.08 (1.38, 4.78) | 3.16 (1.40, 4.92) | 0.812 | 3.10 (1.40, 4.80) | 3.14 (1.44, 4.84) | 0.765 |
|  | APOE | 0.48 (0.12, 0.84) | 0.018 | 0.51 (0.14, 0.88) | 0.43 (0.06, 0.80) | 0.635 | 0.46 (0.08, 0.84) | 0.50 (0.10, 0.90) | 0.781 | 0.47 (0.08, 0.86) | 0.49 (0.10, 0.88) | 0.803 |
| UAP vs. SAP | KNG1 | -1.43 (-2.01, -0.85) | <0.001 | -1.45 (-2.06, -0.84) | -1.40 (-2.04, -0.76) | 0.867 | -1.41 (-2.03, -0.79) | -1.45 (-2.08, -0.82) | 0.854 | -1.42 (-2.05, -0.79) | -1.44 (-2.08, -0.80) | 0.892 |
|  | THBS1 | 0.52 (0.09, 0.95) | 0.018 | 0.54 (0.10, 0.98) | 0.49 (0.04, 0.94) | 0.781 | 0.50 (0.04, 0.96) | 0.54 (0.08, 1.00) | 0.823 | 0.51 (0.05, 0.97) | 0.53 (0.07, 0.99) | 0.798 |
|  | CP | -0.42 (-0.78, -0.06) | 0.026 | -0.44 (-0.82, -0.06) | -0.39 (-0.80, 0.02) | 0.812 | -0.40 (-0.80, 0.00) | -0.44 (-0.84, -0.04) | 0.798 | -0.41 (-0.82, 0.00) | -0.43 (-0.84, -0.02) | 0.823 |
|  | C1R | -5.42 (-7.86, -2.98) | <0.001 | -5.51 (-8.02, -3.00) | -5.28 (-7.92, -2.64) | 0.903 | -5.38 (-7.95, -2.81) | -5.46 (-8.04, -2.88) | 0.916 | -5.40 (-8.00, -2.80) | -5.44 (-8.06, -2.82) | 0.934 |
| AMI vs. UAP | CNDP1 | 3.71 (2.54, 4.88) | <0.001 | 3.75 (2.52, 4.98) | 3.64 (2.33, 4.95) | 0.845 | 3.68 (2.41, 4.95) | 3.74 (2.45, 5.03) | 0.867 | 3.69 (2.40, 4.98) | 3.73 (2.44, 5.02) | 0.856 |
|  | KNG1 | -1.52 (-2.23, -0.81) | <0.001 | -1.55 (-2.30, -0.80) | -1.48 (-2.27, -0.69) | 0.812 | -1.50 (-2.28, -0.72) | -1.54 (-2.32, -0.76) | 0.835 | -1.51 (-2.30, -0.72) | -1.53 (-2.34, -0.72) | 0.848 |
|  | APOL1 | 2.98 (1.84, 4.12) | <0.001 | 3.02 (1.83, 4.21) | 2.91 (1.66, 4.16) | 0.756 | 2.95 (1.72, 4.18) | 3.01 (1.78, 4.24) | 0.802 | 2.96 (1.72, 4.20) | 3.00 (1.76, 4.24) | 0.789 |
|  | KLKB1 | 4.21 (2.95, 5.47) | <0.001 | 4.28 (2.96, 5.60) | 4.09 (2.70, 5.48) | 0.823 | 4.18 (2.84, 5.52) | 4.24 (2.90, 5.58) | 0.856 | 4.19 (2.84, 5.54) | 4.23 (2.88, 5.58) | 0.834 |

Supplementary Table 6. TMT 10-Plex Channel Allocation for Proteomic Analysis

| TMT Batch | Channel 126 | Channel 127N | Channel 127C | Channel 128N | Channel 128C | Channel 129N | Channel 129C | Channel 130N | Channel 130C | Channel 131 |
| --- | --- | --- | --- | --- | --- | --- | --- | --- | --- | --- |
| Batch 1 | HC-01 | HC-02 | HC-03 | SAP-01 | SAP-02 | UAP-01 | UAP-02 | AMI-01 | AMI-02 | Reference |
| Batch 2 | HC-04 | HC-05 | HC-06 | SAP-03 | SAP-04 | UAP-03 | UAP-04 | AMI-03 | AMI-04 | Reference |
| Batch 3 | HC-07 | HC-08 | HC-09 | SAP-05 | SAP-06 | UAP-05 | UAP-06 | AMI-05 | AMI-06 | Reference |
| Batch 4 | HC-10 | HC-11 | HC-12 | SAP-07 | SAP-08 | UAP-07 | UAP-08 | AMI-07 | AMI-08 | Reference |

HC, healthy control; SAP, stable angina pectoris; UAP, unstable angina pectoris; AMI, acute myocardial infarction; TMT, tandem mass tag.
